# Mass Spectrometry Based Imaging of Labile Glucosides in Plants

**DOI:** 10.3389/fpls.2018.00892

**Published:** 2018-06-28

**Authors:** Frederik Bøgeskov Schmidt, Allison M. Heskes, Dinaiz Thinagaran, Birger Lindberg Møller, Kirsten Jørgensen, Berin A. Boughton

**Affiliations:** ^1^Plant Biochemistry Laboratory, Department of Plant and Environmental Sciences, University of Copenhagen, Copenhagen, Denmark; ^2^VILLUM Research Center for Plant Plasticity, University of Copenhagen, Copenhagen, Denmark; ^3^Center for Synthetic Biology, University of Copenhagen, Copenhagen, Denmark; ^4^Metabolomics Australia, School of BioSciences, University of Melbourne, Melbourne, VIC, Australia

**Keywords:** hydroxynitrile glucoside, cyanogenic glucoside, matrix assisted laser desorption ionization, MALDI, *Manihot esculenta*, *Lotus japonicus*

## Abstract

Mass spectrometry based imaging is a powerful tool to investigate the spatial distribution of a broad range of metabolites across a variety of sample types. The recent developments in instrumentation and computing capabilities have increased the mass range, sensitivity and resolution and rendered sample preparation the limiting step for further improvements. Sample preparation involves sectioning and mounting followed by selection and application of matrix. In plant tissues, labile small molecules and specialized metabolites are subject to degradation upon mechanical disruption of plant tissues. In this study, the benefits of cryo-sectioning, stabilization of fragile tissues and optimal application of the matrix to improve the results from MALDI mass spectrometry imaging (MSI) is investigated with hydroxynitrile glucosides as the main experimental system. Denatured albumin proved an excellent agent for stabilizing fragile tissues such as *Lotus japonicus* leaves. In stem cross sections of *Manihot esculenta*, maintaining the samples frozen throughout the sectioning process and preparation of the samples by freeze drying enhanced the obtained signal intensity by twofold to fourfold. Deposition of the matrix by sublimation improved the spatial information obtained compared to spray. The imaging demonstrated that the cyanogenic glucosides (CNglcs) were localized in the vascular tissues in old stems of *M. esculenta* and in the periderm and vascular tissues of tubers. In MALDI mass spectrometry, the imaged compounds are solely identified by their *m/z* ratio. *L. japonicus* MG20 and the mutant *cyd1* that is devoid of hydroxynitrile glucosides were used as negative controls to verify the assignment of the observed masses to linamarin, lotaustralin, and linamarin acid.

## Introduction

Plants synthesize a diverse range of specialized metabolites to fend off herbivores and pests and to communicate with the environment (Møller, 2010; Neilson et al., 2013). In addition, some specialized metabolites like cyanogenic glucosides (CNglcs) may serve as storage and transport forms of reduced carbon and nitrogen used to fine tune primary metabolism (Jenrich et al., 2007; Picmanova et al., 2015; Nielsen et al., 2016; Bjarnholt et al., 2018). Understanding these interactions is key to developing robust crop plants for the future.

Mass spectrometry imaging (MSI) is a powerful technique to guide elucidation of the functional properties of specialized metabolites in nature by enabling determination of their precise cellular localization based on their molecular mass (Shroff et al., 2008; Hamm et al., 2010; Li B. et al., 2011; Sarsby et al., 2012; Li et al., 2013; Becker et al., 2014; Bjarnholt et al., 2014; Hölscher et al., 2014; Boughton et al., 2015; Shroff et al., 2015; Jarvis et al., 2017). Recent technological advances have provided increased sensitivity and improved mass and spatial resolution of their localization (Li et al., 2013; Boughton et al., 2015). Current, approaches to MSI for the spatial measurement of low mass natural products and their metabolites are dominated by Matrix Assisted Laser Desorption Ionisation (MALDI) (Boughton et al., 2015; Boughton and Hamilton, 2017; Weber et al., 2017). For small molecule analysis in complex matrices, the inability to distinguish between the presence of near-isobaric compounds with similar mass-to-charge (*m/z*) ratios provides a significant challenge for mass spectrometry (MS) based identification. Ultra-high mass resolution by MALDI – Fourier Transform Ion Cyclotron Resonance-MS (FTMS) provides significant advantages, using both high mass accuracy to identify the molecular formula (typically less than 2 ppm mass error) and ultra-high mass resolution to resolve metabolites with similar *m/z* (utilizing resolving powers >100,000).

Mapping of labile bio-active natural products to specific cells and tissue-types requires new instrumental and sample preparation methods. This is because the fixation and preparation processes may result in loss or structural degradation of the metabolites, either removing them entirely or decreasing their relative concentrations in the tissue or altering their cellular localisation by diffusion. Hydrolysis of glycosylated natural products during preparation for MALDI-MSI is attributed to the presence of glycosidases which during sectioning and processing are brought into contact with their otherwise compartmentalized substrates as demonstrated for many two component defense systems like those based on CNglcs (Morant et al., 2008). To stabilize tissues between the inherent time difference between sampling and preparation, tissues are generally flash-frozen and stored to retain the structure and distribution of endogenous metabolites prior to analysis. Frozen tissues require cryo-sectioning to generate thin tissue sections which are then generally thaw-mounted directly to a target suitable for analysis. For frozen plant samples containing CNglcs, the cryo-sectioning and freeze-thaw steps disrupt the cellular structures and results in mixing of the CNglcs with β-glucosidase enzymes leading to hydrolysis. This lowers signal intensity and impairs the spatial distribution analyses.

**FIGURE 1:**
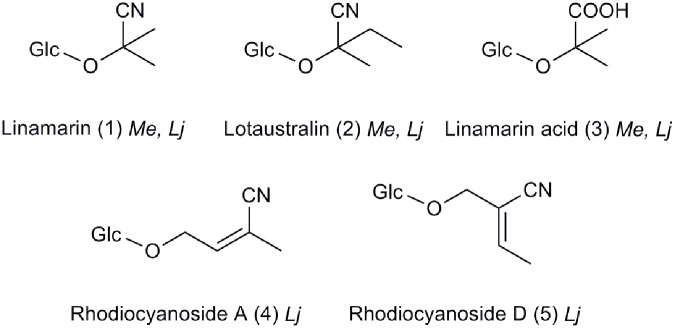
Structures of hydroxynitrile glucosides and their endogenous turn-over products in *Manihot esculenta* (Me) and *Lotus japonicus* (Lj).

Here we present a novel broad based technique to embed, prepare, section, and mount plant tissue suitable for LDI and MALDI-MSI. The technique circumvents hydrolysis and diffusion of labile glycosides. The method was developed using *Manihot esculenta* (cassava) with the aim of being able to accurately monitor the distribution of CNglcs in different tissues at ultra-high mass resolution using FTMS. *M. esculenta* produces the two mono-CNglcs, linamarin (1) and lotaustralin (2), a number of cyanogenic di-glcs and apiosides as well as structurally related amides, acids (3) and anitriles (Picmanova et al., 2015; **Figure 1**), with the latter three being intermediates in the hypothesized endogenous turn-over pathways of CNglcs (Nielsen et al., 2016; Bjarnholt et al., 2018). A second CNglc producing species, *Lotus japonicus*, was used to verify the developed technique. This species produces both linamarin and lotaustralin and the non-cyanogenic hydroxynitrile glucosides rhodiocyanosides A (4) and D (5) (Bjarnholt and Møller, 2008). A non-cyanogenic *L. japonicus* mutant (*cyd1*) harboring a non-functional CYP79A1 enzyme was also included in the present study and provided an experimental negative control system (Takos et al., 2011). Whereas linamarin is the main CNglc present in cassava (Nartey, 1968; Koch et al., 1992; Lykkesfeldt and Møller, 1994), lotaustralin is the main CNglc in *L. japonicus* (Forslund et al., 2004).

**FIGURE 2:**
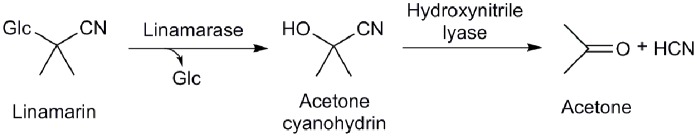
Hydrolysis of the cyanogenic glucoside (CNglc) linamarin in *M. esculenta* and *L. japonicus* as a result of tissue disruption and the action of the β-glucosidase linamarase to form the α-hydroxynitrile, acetone cyanohydrin. Acetone cyanohydrin is dissociated into hydrogen cyanide and acetone by the action of an α-hydroxynitrile lyase. At alkaline pH, the latter process proceeds non-enzymatically.

Cyanogenic glycosides (CNglcs) are specialized metabolites widely distributed throughout the plant kingdom (Gleadow and Møller, 2014). They are α-hydroxynitrile glycosides and as noted above form part of a two-component defense system detonated by a specific β-glucosidase that releases a hydrogen cyanide bomb upon disruption of the cellular structure, e.g., as mediated by a feeding herbivore (**Figure 2**; Morant et al., 2008). CNglcs are formed from amino acids which in the general pathway are converted into α-hydroxynitriles (cyanohydrins) by the action of two membrane bound cytochrome P450s and then via the action of glycosyltransferases converted into mono and di-glycosides (Conn, 1969).

Once considered to function only as components of plant chemical defense systems, it has become apparent that CNglcs contribute to enhance plant (Møller, 2010; Gleadow and Møller, 2014). They show species-specific chemical profiles and patterns of localization, both of which can change during plant ontogeny (Halkier and Møller, 1989; Adewusi, 1990; Koch et al., 1992; Nambisan and Sundaresan, 1994; Nielsen et al., 2016). Little is known about how plants balance biosynthesis, transport, storage, and turnover of CNglcs and how this is influenced by changing environmental conditions. Localisation of CNglcs and related metabolites with high spatial resolution within a range of tissues may be able to help shed light on some of these areas.

## Results

### Sectioning and Embedding Method

The preparation of plant tissues for mass spectrometric imaging required modifications when compared to standard procedures used with animal cells (Norris and Caprioli, 2013; Spengler, 2015). The rigid structure of the plant cell wall, the high intracellular water content and presence of large vacuoles render frozen sections of most plant tissues delicate to handle and prone to fracturing. To bypass this physical property, sections of a thickness above 35 μm were prepared to reduce breakage. For *M. esculenta* stem and tuber samples, section thicknesses of 40 and 60 μm, respectively, offered the best overall analytical details. In the case of *L. japonicus* leaves, a tissue thickness of 40 μm provided the best sections for analysis. Sections were mounted onto glass slides using either a standard ‘freeze-thaw’ approach or a ‘freeze-dry’ approach where frozen sections were transferred onto carbon double sided tape prior to freeze-drying.

Due to the relatively thin and delicate structural nature of leaves, it is not possible to section intact frozen leaves without prior imbedding. Sturdier plant tissues, such as the stems and tubers of *M. esculenta* plants used in this study may be sectioned without imbedding. In this study, we exploited the use of denatured albumin as an inexpensive and readily available embedding medium for fine tissue structures. The leaf material was sandwiched between two pieces of denatured albumin, then gently frozen over a surface of liquid nitrogen and sectioned. This approach offered leaf tissue sections suitable for MALDI-MSI analysis due to the very low background from the albumin, ease of handling and good sectioning properties (Kellersberger et al., 2011, 2012). Albumin contains very few small molecules that could interfere with any MALDI analysis, being made up of water (88–90%), protein (10–12%), and carbohydrate (0.24%) and containing almost no lipid.

### Matrix Deposition Strategies Affect Distribution of Compounds

Matrix selection and deposition is an essential part of MALDI-MSI. 2,5-Dihydroxybenzoic acid (DHB) was chosen as matrix since it has been shown to work well with low molecular mass molecules such as nucleotides, peptides, lipids, and saccharides in positive ion mode (Zaima et al., 2010). Two deposition technologies were tested: Spray (wet) and sublimation (dry).

**FIGURE 3:**
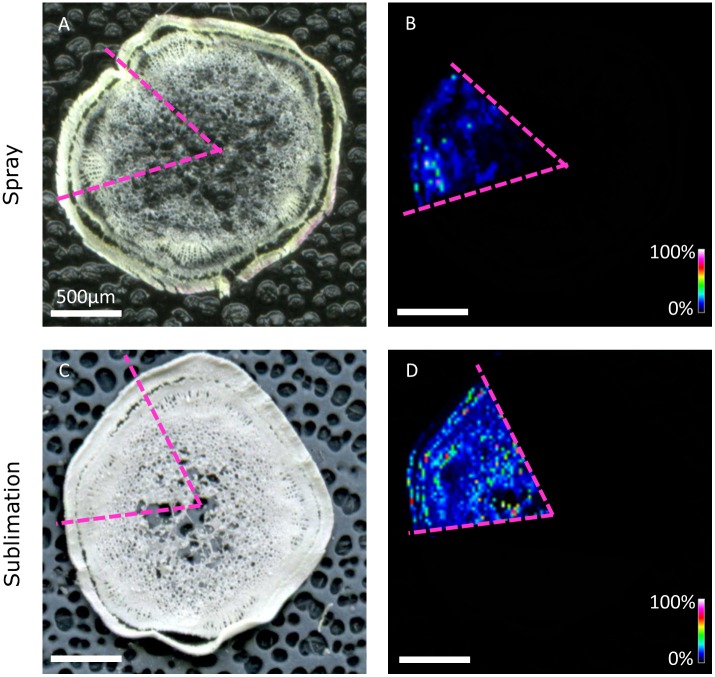
Distribution of linamarin ([M+K]+ *m/z* 286.06873) in 40 μm sections of a *M. esculenta* stem excised approximately 5 cm from the apex and covered with 2,5-dihydroxybenzoic acid (DHB) either by TM spray **(A,B)** and sublimation **(C,D)**. Data was not normalized, Scale represents 0–100% signal intensity. Bar: 500 μm.

Sections were first mounted using a freeze-dry method and analyzed by MALDI-MSI. Initially, we focussed on measuring the distribution of linamarin reported as the most abundant CNglc in the *M. esculenta* tissue using wide mass range profiling (Nartey, 1968; Lykkesfeldt and Møller, 1994; Selmar, 1994). Linamarin was predominantly observed as the potassium salt [M+K]^+^ at *m/z* 286.06873 (calc. 286.06875, 0.05 ppm mass error) with a relatively low response compared with other endogenous metabolites (data not shown). The presence of the potassium salt agrees with previous findings (Kebarle, 2000; Kristensen et al., 2005). In the vacuole, the concentration of potassium ions may reach 500 mM (de Lacerda et al., 2003; Kanto et al., 2012). To increase sensitivity for linamarin, a method using Continuous Accumulation of Selected Ions (CASI) scan across a narrow *m/z* range was employed to filter highly abundant ions at different *m/z*. Vast differences were observed in linamarin response and localisation when comparing sections with DHB applied by either spray or sublimation (**Figure 3**). Specifically, results from the sprayed section showed a higher degree of tissue disruption and poor resolution of linamarin distribution, including very low signal (if any) in comparison to the signal obtained in equivalent areas of tissues when the matrix was applied by sublimation (**Figure 3**).

### Sample Mounting Techniques Affect Metabolite Stability, Intensity, and Distribution

**FIGURE 4:**
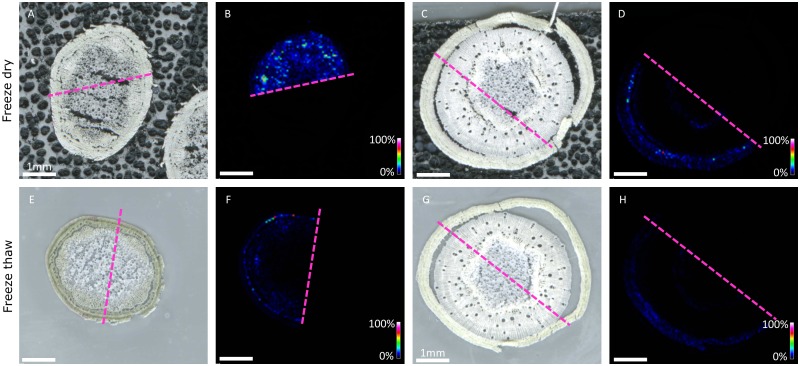
The linamarin ([M+K]+ *m/z* 286.06873) signal obtained in 40 μm cross sections of *M. esculenta* stem excised 5 cm below apex **(A,E)** or 5 cm above soil surfaced **(C,G)** mounted either by freeze-dry **(A–D)** or freeze-thaw methodology **(E–H)**. Scale represents 0–100% normalized signal intensity.

**FIGURE 5:**
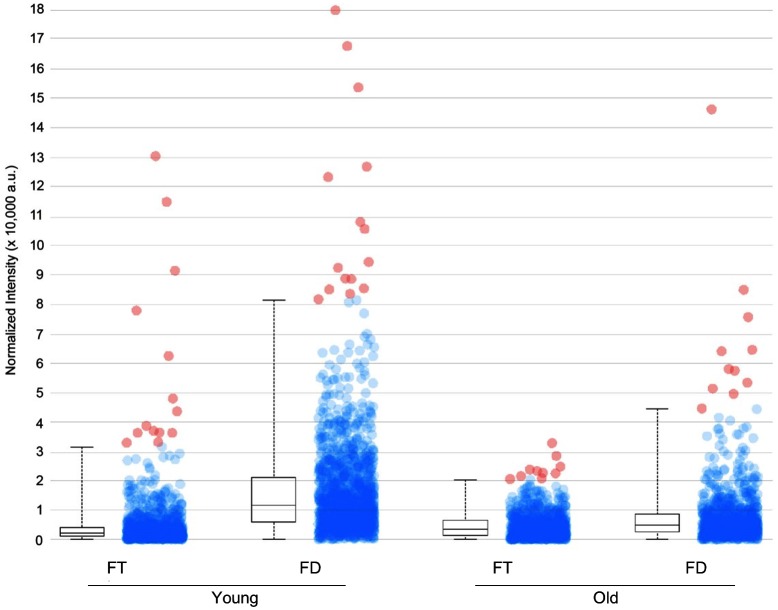
Box and dot plot of normalized linamarin ([M+K]+ *m/z* 286.06873) signal intensities in young and older 40 μm cross sections of a cassava stem mounted either by the freeze-thaw method (FT) or freeze-dry method (FD). Red dots represent outliers.

In *M. esculenta*, hydrolysis of the CNglcs linamarin and lotaustralin is catalyzed by linamarase, which is a highly stable β-glucosidase (Elias et al., 1997). To determine the stability and dislocation of CNglcs during the section mounting process, two different mounting techniques were compared, ‘freeze-thaw’ and ‘freeze-dry’ using sequential sections from the same tissue sample. For the mounting techniques, a standard freeze-thaw mounting to glass slide followed by vacuum drying was compared to mounting a frozen section to carbon double-sided tape followed by freeze-drying. In both treatments, sublimation with the matrix DHB was carried out before CNglc analysis. The experimental material used was *M. esculenta* stem tissues from young parts of the *M. esculenta* plant excised 5 cm from the apex and from older parts excised 5 cm above the soil surface. Images of freeze-dried vs freeze-thaw mounted sections on both young and older stem tissues were collected in the same analytical instrument sequence to compare the localisation of linamarin across the stem using the two different mounting techniques (**Figure 4**). Results showed that the standard freeze-thaw mounting approach led to an overall twofold to fourfold decrease in linamarin ion intensity when compared to the freeze-dried carbon tape based method in similar areas of tissue (**Figure 5**). Overall, the results indicate that a significant amount of the CNglc content is lost using the freeze-thaw mounting technique compromising to some extent the ability to accurately assess the distribution of linamarin in the tissue section.

### Distribution and Cyanogenic Compounds in *M. esculenta*

**FIGURE 6:**
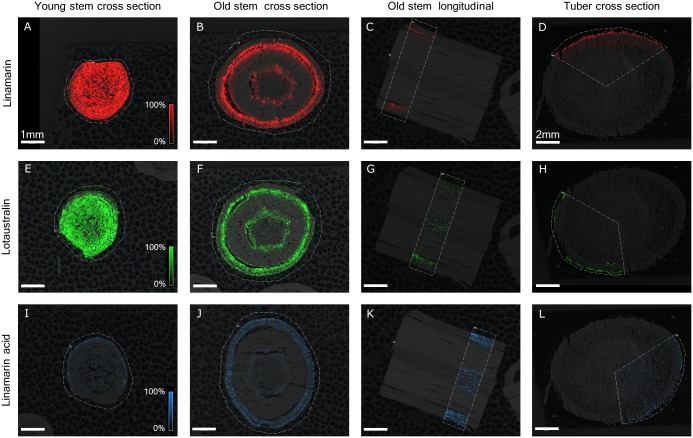
Distribution of linamarin ([M+K]+ *m/z* 286.06873) **(A–D)**, lotaustralin ([M+K]+ *m/z* 300.08435) **(E–H)**, and linamarin acid ([M+K]+ *m/z* 305.06326) **(I–L)** in 40 μm sections of young (5 cm from apex) and old (5 cm from soil surface) *M. esculenta* stem section and in 60 μm sections of tuber, all mounted using the freeze-dry method and sublimated with 2,5-DHB. Bars indicate 1 mm for all stem sections and 2 mm for all tuber sections. Scale represents 0–100% normalized signal intensity.

*Manihot esculenta* stem sections from the upper and lower part of the plant and from tubers proved to be useful proof of concept samples for visualizing the differential distribution of linamarin, lotaustralin [M+K]^+^ *m/z* 300.08435 (calc. 300.08439, 0.15 ppm error) and linamarin acid [M+K]^+^ *m/z* 305.06326 (calc. 305.06332, 2.16 ppm error). Linamarin and lotaustralin were observed to be present and evenly distributed throughout all tissue types of the young stem material except that the concentrations were somewhat reduced in the central core. In the sections from the older part of the stem, linamarin and lotaustralin were restricted to the first cell rows of the cortex, to cells around the phloem and to the parenchymatous cells surrounding the secondary xylem in the center of the stem (**Figure 6**). For tubers, linamarin and lotaustralin was localized to the periderm and vascular ring. Linamarin acid was found predominantly in the epidermis of stems and was detectable in the entire tuber but predominantly in the periderm. Cross-sections and longitudinal sections of old stems showed the same spatial distribution.

### Testing of the Sample Preparation Technique and Observed Masses in *Lotus japonicus*

**FIGURE 7:**
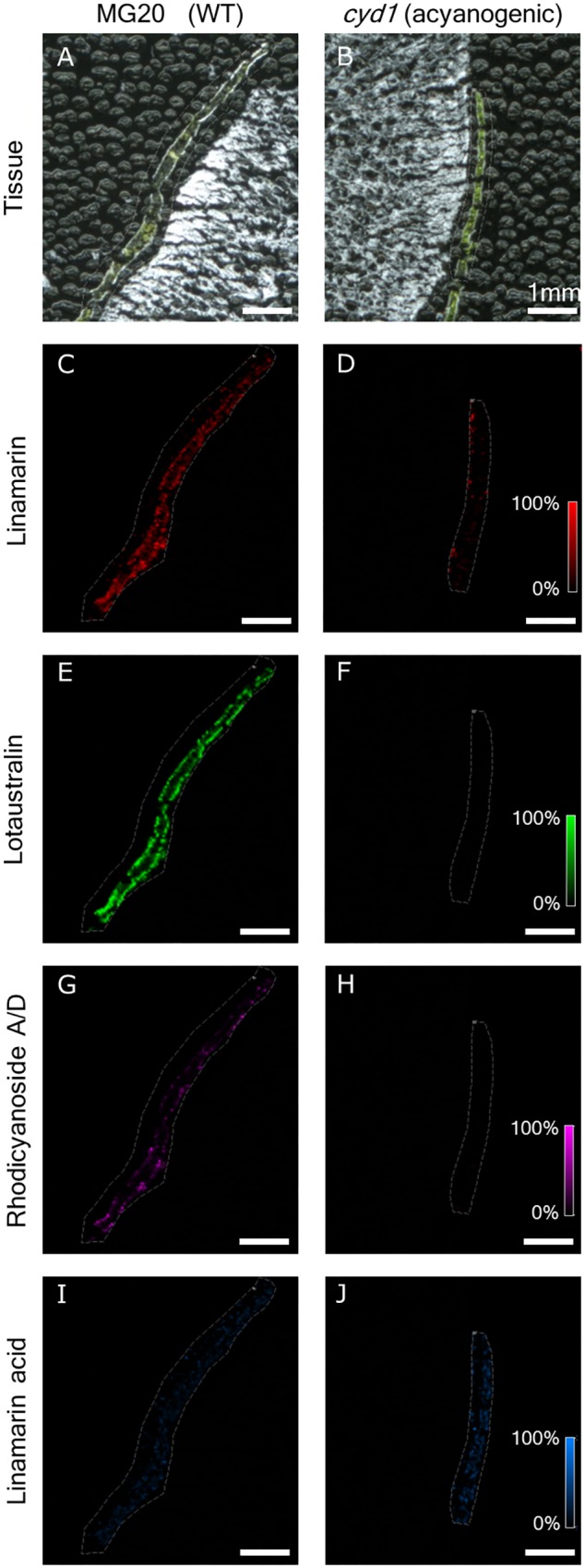
Presence of linamarin ([M+K]+ *m/z* 286.06873), lotaustralin ([M+K]+ *m/z* 300.08435), rhodiocyanoside A/D ([M+K]+ *m/z* 298.06944), and linamarin acid ([M+K]+ *m/z* 305.06326) in 40 μm cross sections of Lotus japonicus MG20 **(A,C,E,G,I)** and cyd1 **(B,D,F,H,J)** leaves mounted using the freeze-dry method and sublimated with 2,5-DHB. Scale represents 0–100% normalized signal intensity.

**FIGURE 8:**
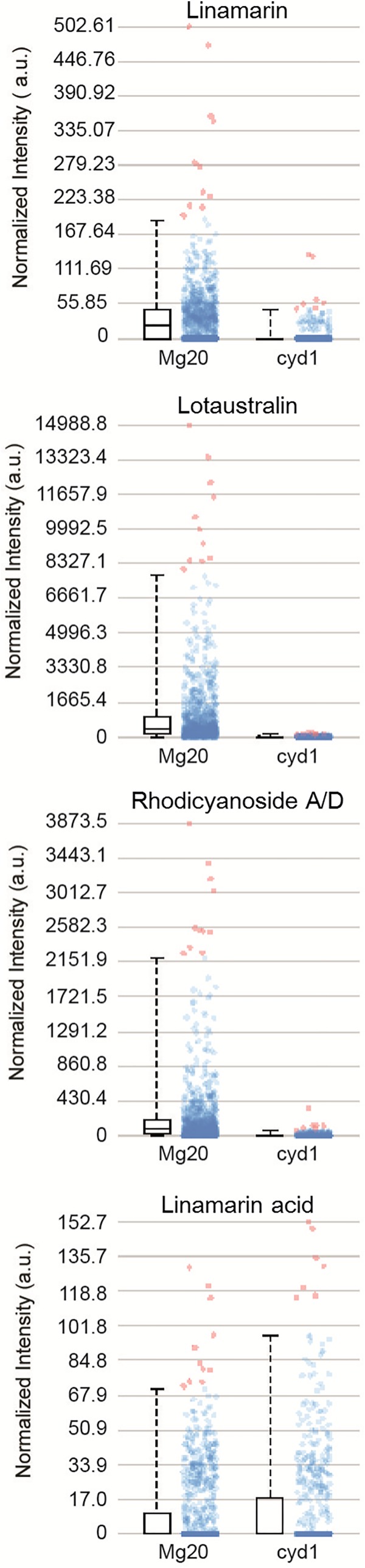
Box and dot plot of normalized signal intensities of linamarin ([M+K]^+^ *m/z* 286.06873), lotaustralin ([M+K]^+^ *m/z* 300.08435), rhodiocyanoside A/D ([M+K]^+^ *m/z* 298.06944), and linamarin acid ([M+K]^+^ *m/z* 305.06326) in 40 μm cross sections of *L. japonicus* MG20 and *cyd1* leaves mounted using the freeze-dry method and sublimated with 2,5-DHB. Red dots represent outliers.

Once the stable MALDI-MSI preparation and sampling method, utilizing the denatured albumin embedding and freeze-dry method was established for *M. esculenta*, the techniques were tested on *L. japonicus* tissue. In the wild-type, CYP79D3 catalyzes the first committed step in CNglc biosynthesis in the leaves (Forslund et al., 2004). The *cyd1* mutant of *L. japonicus* does not produce CNglcs because it contains a non-functional CYP79D3 (Takos et al., 2010). *L. japonicus* MG20 wildtype plants and *cyd1* mutants were analyzed for ions expected to be the potassium adducts of lotaustralin, linamarin, and rhodiocyanoside A/D (observed [M+K]^+^ *m/z* 298.06944, calc. 298.06874, 2.33 ppm error). The signals for these adducts were observed in wild-type plants but were absent in the *cyd1* mutant, thus linking these ions to the *in vivo* presence of their respective metabolites (**Figures 7, 8**). Linamarin, lotaustralin, and rhodiocyanoside A/D appeared to localize to the epidermal layers on both sides of the leaf. To further understand the role of the acid forms of CNglcs, the localisation of linamarin acid and lotaustralin acid were examined. An ion corresponding to the potassium adduct of linamarin acid [M+K]^+^ *m/z* 305.06519 (calc. 305.0639, 4.23 ppm mass error) was observed in both wild-type and *cyd1*. The respective signal for the potassium adduct of lotaustralin acid was not observed.

## Discussion

The classically assigned physiological function of CNglcs is their action as defense compounds through cyanogenesis. The defense response is activated when otherwise compartmentalized CNglcs and hydrolytic enzymes are brought into contact by tissue disruption (Morant et al., 2008). More recently CNglcs have been attributed functions as transport and storage forms of reduced nitrogen and as ROS scavengers (Møller, 2010; Picmanova et al., 2015; Nielsen et al., 2016; Bjarnholt et al., 2018), resulting in the transient formation of the corresponding amides and carboxylic acids. Spatial analysis by MSI may guide the understanding of the orchestration of such functions at the tissue and cellular level and result in the discovery of hitherto unrecognized new functionalities.

**FIGURE 9:**
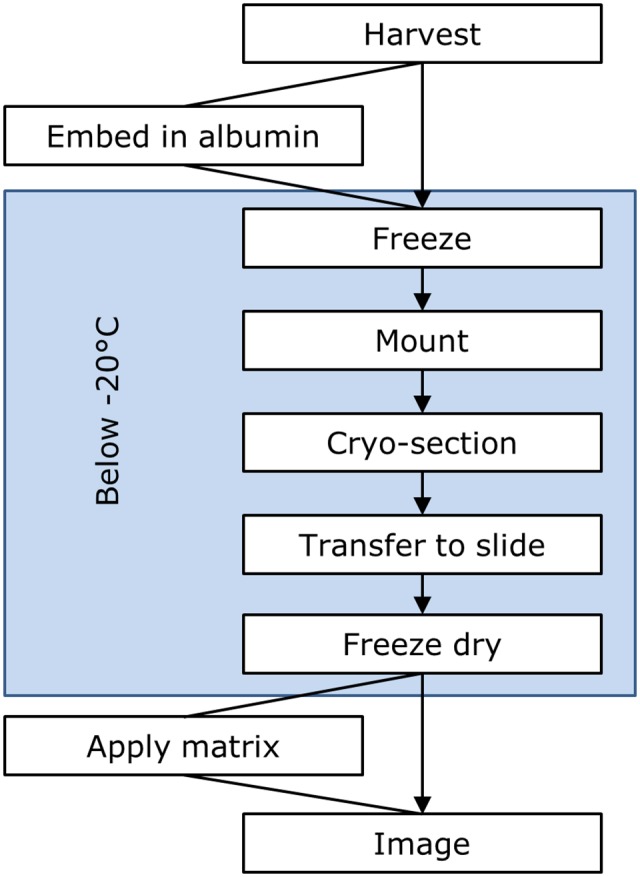
Workflow diagram of preparation of tissue containing labile compounds for mass spectrometry based imaging.

The separation of CNglcs and hydrolytic enzymes is partly disrupted upon tissue sectioning. In an aqueous cellular environment, the β-glycosidases are active and may by simple diffusion processes gain contact with and hydrolyse CNglcs present. A recent report on MSI of CNglcs in flax (*Linum usitatissimum*) seed noted the potential for rapid hydrolysis of CNglcs but no measures to prevent this were provided (Dalisay et al., 2015). We observed that the mounting technique and method for application of matrix had a significant impact on signal intensity and localisation of CNglcs in *M. esculenta* tissues and we suggest keeping tissues frozen during all steps from sampling until matrix application (**Figure 9**).

Denatured albumin has previously been used in a number of different applications including quantitation directly from images (Kellersberger et al., 2011, 2012). A typical histological approach involves embedment of tissues in an Optimal Cutting Temperature (OCT) compound prior to freezing. OCT compounds have been shown to smear across the surface of tissues during cryo-sectioning and to significantly suppress analyte signal (Schwartz et al., 2003). Other embedding media including agar (Marques et al., 2014), gelatine (Ye et al., 2013; Horn et al., 2014; Korte and Lee, 2014), and carboxymethylcellulose (Yoshimura et al., 2012; Bencivenni et al., 2014) are available and have been employed for a variety of different tissue types. In general, aqueous gels or solutions have a tendency to spread or wet surrounding tissue slices when thawed, potentially reducing the spatial information obtained and inducing the hydrolysis of labile compounds. We found that handling the tissues while frozen and the inclusion of a freeze-dry step circumvented these negative effects. Inclusion of a freeze-dry step is recommended and would mitigate wetting if using a gel based embedding medium. More recently, methods using frozen gel embedding mediums and cryotape transfer have been demonstrated in barley and quinoa seeds but require specific cryotape (Gorzolka et al., 2016; Jarvis et al., 2017). The denatured albumin embedding, section and freeze-dry method described here presents a simple and low-cost alternative to the sample handling procedures described above.

In a single scan of a tissue sample, full range unbiased MSI offers an unprecedented account of the metabolic status with spatial resolution reaching the cellular level. In the event of low signal intensity for a desired analyte, the advanced features of the Bruker SolariX system enable targeted CASI experiments and trapping, increasing the sensitivity for individual metabolites. The CASI based method applies a quadrupole filter thus excluding competing ions from the detection cell. When coupled to high mass resolution, the approach provides excellent analyte signal intensity and capability to measure distribution of specific metabolites of interest. This was documented in the studies of the *M. esculenta* tissue samples, where the CASI based methods provided the best result by excluding competing ions from the detection cell increasing the sensitivity for the specific metabolites in question greatly, particularly when testing different mounting methods.

MALDI-MSI results for *M. esculenta* tubers demonstrated that linamarin and lotaustralin are localized in the periderm and vascular ring tissue (**Figure 6**). This pattern corresponds to earlier findings using DESI-MSI on fresh sections and orthogonal LC-MS on laser dissected tissues in which these CNglcs were also found to be localized in these regions (Li et al., 2013). Extending these previous finding, we show for the first time the localisation of the CNglcs linamarin and lotaustralin in stems and the presence of the endogenous turn-over product linamarin acid in stems and tubers of *M. esculenta*. Localisation of CNglc pathway transcripts and enzymes in *M. esculenta* stems have shown the pathway to localize to vascular tissue, cortex, and around the laticifers (Kannangara et al., 2011). The same localisation patterns were found for linamarin and lotaustralin in the data presented here (**Figure 6**). Linamarin acid is predominant in the sections obtained from the older part of the stem, which corresponds to the observations reported by Picmanova et al. (2015). Other intermediates of the proposed CNglc turnover pathway were not found but are generally present in very low concentrations (Picmanova et al., 2015), potentially below the limits of detection within this experiment. Glucosinolates—sulfur containing defense compounds found in Brassicaceae species—share similar initial biosynthetic steps and mode of bioactivation (Bak et al., 1998; Ishida et al., 2014). Localisation studies of glucosinolate biosynthesis and sulfur in stems of *Arabidopsis thaliana* linked both to specific cells in the vascular tissues (Koroleva et al., 2000; Li J. et al., 2011). The similar localisation patterns for glucosinolates and CNglcs in the vascular tissue might indicate they share even more functional parallels. The localisation of linamarin and lotaustralin to the first row of cortex cells and the vascular tissue in the older parts of the stem could offer protection against both generalist herbivores and phloem feeders.

In MSI, compounds are observed as their respective charged ions and are then first putatively annotated by the accurate mass match and the isotopic peak profile. Due to the possibility of many metabolites possessing a similar mass, an orthogonal technique, such as tandem-MS directly off tissue, LC-MS, NMR or the use of prior information are required to confirm identity (Boughton et al., 2015; McDonnell et al., 2015; Dias et al., 2016). The presence of linamarin, lotaustralin and linamarin acid have previously been identified using various orthogonal techniques in both *M. esculenta* and *L. japonicus* (including rhodiocyanosides A/D) (Forslund et al., 2004; Picmanova et al., 2015; Nielsen et al., 2016; Bjarnholt et al., 2018), but their spatial distributions have not been visualized using MALDI-MSI. In *L. japonicus*, both a cyanogenic wild-type and an acyanogenic mutant were available to test the sample preparation technique developed with *M. esculenta* and to ensure that the observed masses correspond to the CNglcs of interest and their related metabolites. A simple comparison between the two types can provide a clear statement (presence or absence) whether the observed mass indeed correspond to the metabolite of interest. One peculiar observation was the presence of linamarin acid in both wild-type and the acyanogenic *cyd1* mutant. This indicates that linamarin acid may be formed by an additional route not related to endogenous turn-over of linamarin.

The presented method provides a broadly applicable preparation technique that enables imaging of plant metabolites using MALDI-MSI. For labile metabolites acted upon by hydrolysing enzymes it was found essential to halt enzymatic action by freeze-drying. Likewise, dry sublimation based matrix deposition significantly increased the metabolite signal above the signal obtained using wet spray based matrix deposition techniques. High mass accuracy, complementary LC-MS data and the availability and use of biological control samples like cyanogenic and acyanogenic cultivars provide independent coupling of *m/z* values observed to the known presence or absence of the labile metabolites. The advanced features of the MALDI-FT-ICR-MSI allowed targeted analysis of plant tissue metabolites with low abundancy, which would otherwise be difficult to detect. MSI is a potent new technology but cannot stand alone in structural identification of the constituents present in the samples analyzed. Control images obtained with reference compounds added to the dried tissue sample offers an alternative to the use of mutants. Appropriate sample preparation methodologies need to be adhered to in order to gain trustworthy results.

## Experimental Procedures

### Plant Material

*Manihot esculenta* (cassava) cultivar MAus7 (kindly provided by Professor Roslyn Gleadow, Monash University, Melbourne) was propagated from stem cuttings and grown in a greenhouse at a 30°C/16 h day and 20°C/8 h night regime. *L. japonicus* MG20 and *cyd1* mutant seeds were provided by Dr. Daniela Lai and the plants grown in a greenhouse at 21°C day/16 h and 18°C night/8 h regime.

### Tissue Preparation

*Manihot esculenta* stem (‘young’ tissue: 5 cm from apex, ‘old’ tissue: 5 cm above soil with stem diameters of approximately 4 and 8 mm) and tuber (with approximately diameter of 12 mm) tissues were harvested directly from 3 to 5 months old plants and immediately frozen in a 50 mL Falcon^®^ tube placed in liquid nitrogen. Commercial chicken eggs were boiled and peeled. The denatured egg white composed of albumin was collected. Suitably sized pieces were then cut to size for embedding the leaf sections. *L. japonicus* leaf samples were harvested directly from the plant and embedded in a 2 cm cube of denatured albumin (boiled egg-white). The albumin block was wrapped in cellophane and frozen gently by placing it directly above a liquid nitrogen surface. Tissues and the frozen blocks were mounted to a chuck using Tissue-Tek^®^ Optimal Cutting Temperature (O.C.T.) compound. Sections were cut at 35–60 μm thickness making sure no OCT compound came into contact with the sectioned tissue. For freeze-thaw mounting, sections were transferred onto pre-chilled (kept in the cryomicrotome at -20°C) Menzel–Gläser Superfrost Ultra-Plus Glass slides then gently warmed from the rear of the slide by pressing a finger until the glass was warm enough for the section to thaw and attach to the slide. For freeze-dried mounting, sections were transferred to pre-chilled glass slides with mounted carbon double-sided tape (ProSciTech, Thuringowa, QLD, Australia) and gently pressed onto the carbon double-sided tape using a fine paintbrush. All slides prepared with sections were kept frozen in a 50 mL Falcon^®^ tube placed in liquid nitrogen and were freeze-dried overnight. Mounted sections of both techniques were then stored in a vacuum-desiccator at room temperature.

### Matrix Deposition

To compare the different matrix deposition strategies, mounted tissue sections were covered with 2,5-DHB (Sigma-Aldrich) using either wet (spray) or dry (sublimation). Wet matrix deposition was carried out using a HTX TM-Sprayer (HTX Industries, Chapel Hill, NC, United States) fitted with a Shimadzu LC20-AD HPLC pump (Shimadzu Australia, Rydalmere, NSW, Australia) using the following settings: Flow rate: 0.1 mL/min; Gas flow rate: 10 L/min; Nozzle temperature: 30°C; Spray conditions: 8 passes, 900 mm/min, spacing of 2 mm with alternate passes at a 90° offset and repeat passes set to an offset of 1 mm. DHB was dissolved in ethanol (100%) at a concentration of 10 mg/mL providing a matrix concentration of xx mg/mm^2^. For dry application, DHB was sublimed onto tissue sections using a custom built sublimation apparatus at temperatures of 130–140°C at vacuum pressures less than 0.1 mBar for a period of 7–8 min to generate a matrix coverage of 0.3 ± 0.1 mg/cm^2^.

### Mass Spectrometer

For spatial mass spectrometric analysis, a Bruker (Bruker Daltonik, Bremen, Germany) SolariX XR 7 Tesla Hybrid ESI/MALDI-FT-ICR-MS was used. The instrument was operated in the positive ion mode using optimized instrumental settings. For full scan experiments, a mass range from *m/z* 100–800 was employed with analysis instrument set to broadband mode with a time domain for acquisition of 2M providing an estimated resolving power of 130,000 at *m/z* 400. For targeted analysis, CASI was used with a center mass set to *m/z* 286.1 (linamarin [M+K]^+^), *m/z* 300.1 (lotaustralin [M+K]^+^) and *m/z* 305.1 (linamarin acid [M+K]^+^) with 1 Da window chosen using a total of 1 ICR cell fills. The laser was set to 28 or 30% power using the minimum spot size resulting in ablation spots of approximately 35–40 μm in diameter. A total of 1,500–2,000 shots were fired per spectrum at a frequency of 2 kHz within a 50 μm × 50 μm array. Optical images of tissue sections were acquired using an Epson Photosmart 4480 flatbed scanner using a minimum setting of 4,800 d.p.i.

### Data Analysis

Acquired mass spectrometry data were analyzed using Compass FlexImaging 4.1 (Bruker) and were normalized to Total Ion Chromatogram (TIC) and hierarchical cluster analysis conducted using a peak tolerance of 2 ppm mass error. Individual spectra were analyzed using Data Analysis 4.2 with peak lists generated using signal to noise threshold = 4 and 0.15% base peak height threshold. For quantification purposes similar areas of tissue (cell type and total area) were selected by generating specific regions of interest (ROI) using FlexImaging software. The ROI were exported as a spectrum list and imported into Data Analysis where an average spectrum was generated for each specific ROI. The average signal intensity of the respective *m/z* was then compared. Data were imported into SCiLS lab software with the respective ROIs and Box-dot plots were generated.

## Author Contributions

FBS, BB, BLM, and KJ planned the project and the experiments. FBS, AH, KJ, and BB conceptualized and developed the methods. FBS collected and prepared the samples for analysis. FBS and BB analyzed the samples, interpreted the results, and drafted the manuscript. BB and DT did additional data analysis. FBS, BB, KJ, AH, and BLM commented on the manuscript for final version.

## Conflict of Interest Statement

The authors declare that the research was conducted in the absence of any commercial or financial relationships that could be construed as a potential conflict of interest.
